# Anabolic androgenic steroids and carcinogenicity focusing on Leydig cell: a literature review

**DOI:** 10.18632/oncotarget.24767

**Published:** 2018-04-10

**Authors:** Monica Salerno, Orazio Cascio, Giuseppe Bertozzi, Francesco Sessa, Antonietta Messina, Vincenzo Monda, Luigi Cipolloni, Antonio Biondi, Aurora Daniele, Cristoforo Pomara

**Affiliations:** ^1^ University of Foggia, Department of Clinical and Experimental Medicine, Foggia, Italy; ^2^ University of Catania, Department of Medical, Surgical and Advanced Technologies, “G.F. Ingrassia”, Catania, Italy; ^3^ University of Campania “L. Vanvitelli”, Department of Experimental Medicine, Naples, Italy; ^4^ Università degli Studi di Roma “La Sapienza”, Department of Public Health, Roma, Italy; ^5^ University of Catania, Department of Surgery, Catania, Italy; ^6^ University of Campania “L. Vanvitelli”, CEINGE Biotecnologie Avanzate S.C. a r.l., Naples, Italy

**Keywords:** abuse, anabolic-androgenic steroids (AAS), carcinogenicity, insulin-like growth factor 1 (IGF-1), molecular mechanisms

## Abstract

Anabolic androgenic steroids (AAS) are some of the most common drugs used among athletes, frequently in combination with resistance training, to improve physical performance or for aesthetic purpose. A great number of scientific reports showed the detrimental effects of anabolic androgenic steroids on different organs and tissues. In this literature review, we analyzed the AAS-mediated carcinogenicity, focusing on Leydig cell tumor.

AAS-induced carcinogenicity can affect DNA transcription through two pathways. It can act directly via the androgen receptor, by means of dihydrotestosterone (DHT) produced by the action of 5-a-reductase. It can also work through the estrogen receptor, by means of estradiol produced by CYP19 aromatase. In addition, nandrolone and stanazolol can activate the PI3K/AKT and PLC/PKC pathways via *IGF-1*. This would result in cell proliferation in Leydig cell cancer, or magnify cyclin D1 concentration inducing breast cell proliferation.

AAS abuse is becoming a serious public health concern in view of the severe health consequences secondary to AAS abuse. The negative role of AAS in supraphysiological dosage impairs the expression of enzymes involved in testosterone biosynthesis. Abnormal synthesis of testosterone plays has a negative effect on the hormonal changes/regulation, and might be involved in certain carcinogenic mechanisms. At the light of this review, it could become very interesting to perform an information campaign more strengthened in gyms and schools in order to prevent male fertility impairment and other tissues damage.

## INTRODUCTION

Anabolic androgenic steroids (AAS) are one of the most commonly used drugs among athletes to improve physical performance. The use of AAS was prohibited by the International Olympic Committee in 1976 and more recently the World Anti-Doping Agency included these compounds in the list of prohibited substances [1]. Despite those bans, AAS abuse is continuing to increase particularly in the general population at fitness centers, mainly for aesthetic purposes [1–3]. The non-medical use of AAS among athletes and specific subsets of the general population (high school and college students) is considered a major and widespread public health issue [4–6]. It is becoming increasingly clear that the abuse of AAS is associated with serious adverse effects to the liver [7], cardiovascular [8, 9], central nervous [10–12], musculoskeletal [13–15], endocrine [11], fertility and reproductive [16–18] systems.

AAS are synthetic derivatives of testosterone and their pharmacodynamics is similar to all other steroid hormones. AASs are membrane-permeable and influence the nucleus of cells by direct action. When the exogenous hormone penetrates the membrane of the target cell, the first step is the link to an androgen receptor (AR) located in the cytoplasm of the cell. From there, the compound hormone-receptor diffuses into the nucleus, where it either alters gene expression [19], or activates processes that send signals to other parts of the cell [20]. AASs exert their actions by several different mechanisms: (i) they modulate androgen receptor expression as a consequence of intracellular metabolism; (ii) they affect directly the androgen receptor and thus subsequent interaction with co-activators and transcriptional activity; (iii) they interfere with glucocorticoid receptor expression eliciting an anticatabolic effect; and (iv) they act on the CNS resulting in behavioral changes, following the genomic and non-genomic pathways [21].

Thanks to drug designers, to date, more than 100 AAS compounds were synthesized. Analyzing their chemical structure, metabolic half-life, and physiological effects, three classes of AAS can be identified. The first class was obtained by the esterification of the 17β-hydroxyl group of testosterone and includes testosterone propionate and testosterone cypionate. The second class was composed by AASs esterified, connected with the long side chain moieties, with a substitution of a hydrogen for the methyl group at C19. The third class of AAS comprises those compounds that are alkylated at C17, such as 17α-methyltestosterone and stanozolol [22].

Many animal and “*in vitro*” studies have demonstrated that supraphysiologic doses of AAS enhance the expression of oxidative stress proteins as well as of inflammatory and proapoptotic mediators [23]. To the best of our knowledge, only a few studies have been focused on the specific effect of ASS on carcinogenicity.

A relationship between AAS use and cancer was previously suspected and recently proven. Clinical cases reported a causal connection between AAS use and both hepatocellular adenomas and adenocarcinomas. The physiopathological mechanism hidden behind this connection remains unclear. What is known is that the AAS induced biological effect is carried out by the androgen receptor (AR), an intracellular receptor located not only into reproductive organ cells but also into bone, muscle, brain, liver, kidney cells and adipocytes. AAS-AR binding initiates a cascade of events: receptor dimerization, nuclear translocation, binding to the specific promoter of genetic sequences and expression modifications [24].

## FROM AAS GENOTOXICITY TO CARCINOGENICITY

**Figure 1:**
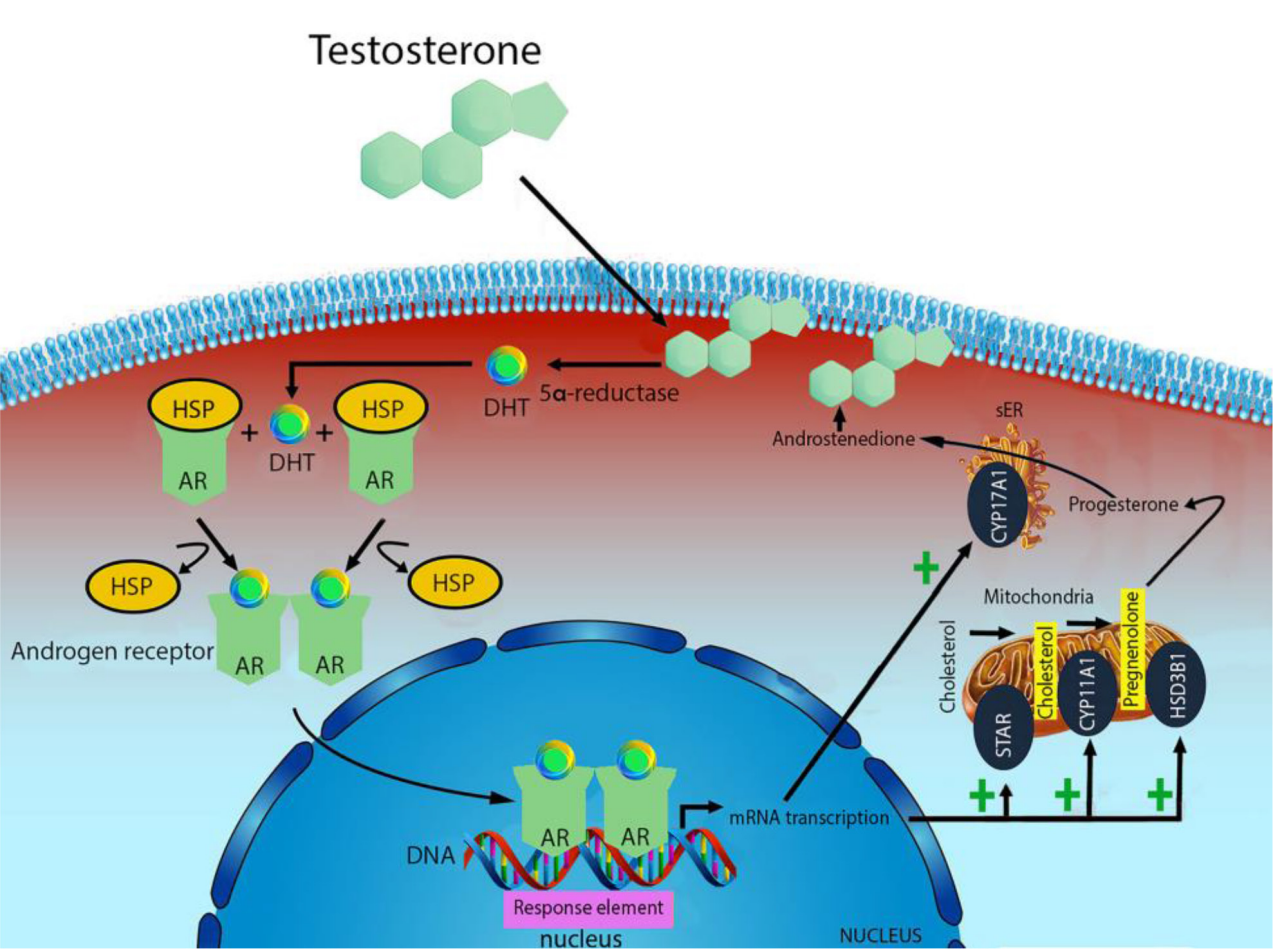
Mechanism of testosterone action Free testosterone is transported into target tissue cell cytoplasm, where it can either bind the androgen receptor, or be reduced to 5α-di-hydro-testosterone (DHT) by the cytoplasmic enzyme 5-alpha reductase. The T-receptor complex undergoes to a structural change that allows its translocation into the cell nucleus, where it directly binds to specific nucleotide sequences of the chromosomal DNA. The produced mRNA promotes the biosynthesis of testosterone.

Testosterone is mainly produced by the Leydig cells of testes in males, and ovaries and theca cells in females [25–26]. Smaller amounts are also synthesized by the adrenal gland in both sexes [27]. The production is regulated by a complex neuroendocrine mechanism which includes the pulsatile release of luteinizing hormone (LH) and subsequent cAMP activation of steroidogenic cascade and numerous steroidogenic stimuli and intratesticular factors that play a role in the intricate regulatory network of testosterone [28]. Cholesterol is the common substrate for all steroid hormones biosynthesis which is completed in the mitochondria. Steroidogenic acute regulatory protein (STAR) transfers cholesterol to the inner membrane of mitochondria. Through the mobilization and delivery from the outer to the inner mitochondrial membrane, cholesterol is converted to pregnenolone by the cytochrome P450 cholesterol side-chain cleavage enzyme (CYP11A1, also known as P450scc) [29]. Pregnenolone is further metabolized to progesterone by mitochondrial or microsomal 3b-hydroxysteroid dehydrogenases (HSD3B1). In Leydig cells, maturation of progesterone to androstenedione is catalyzed by the 17a-hydroxylase/C17–20lyase (CYP17A1 also known as P45017A1); further conversion of androstenedione to testosterone is dependent on the activity of 17b-hydroxysteroid dehydrogenase (17bHSD), steroid dehydrogenase specific for androgen production [27, 30] (Figure 1).

There are three main pathways in which testosterone exerts its effects. It can act directly on androgen receptor, or via dihydrotestosterone (DHT) produced by the action of 5-a-reductase, or via estrogen receptor by means of estradiol produced by CYP19 aromatase [31]. *NR3A (*also known *GRIN3A*) is an androgen nuclear receptor normally linked to heat shock proteins (HSP). When testosterone and DHT, bind to this receptor in the presence of bio-available androgens, it undergoes a conformational change, making free-form HSP, dimerization and moving through the nucleus. Here, this can activate DNA transcription of specific responsive genes through the DNA-binding domains to androgen response elements (ARE), by means of its zinc-finger motif. [32].

AAS mimics testosterone physiological effect, by inducing expression alterations on of DNA sequences. For example, Nandrolone is transported into the target tissue cell cytoplasm as testosterone. However, the mRNA produced interferes with the physiological biosynthesis of testosterone, reducing cholesterol conversion to pregnenolone through CYP11A1. Analogously, the conversion from progesterone to androstenedione through CYP17A1 is reduced resulting in diminishing endogenous testosterone production [33].

However, the genotoxicity mechanism is not well understood. Torres-Bugarín *et al.* [34], in a review analyzing androgen effects on cellular functions, concluded that a combination of genetic and epigenetic factors is the cause of toxicity, mutagenicity, genotoxicity, and carcinogenicity of sexual hormones. Epigenetic factors include three molecular mechanisms, controlling genetic transcription: DNA methylation, histone modifications and chromatin condensation [35]. DNA methylation inhibits the bind between transcriptional factors and their target sequences, both promoters, and introns, impeding the activation of transcriptional expression. In an animal model, *5α-DHT* increased DNA methylation of *srd5α2* (steroid 5α-reductases type 2) [36]. The latter is also regulated by chromatin condensation degree [37].

On taking account genetic factors, testosterone synthetic derivatives can be metabolized to 17β-estradiol in adipose, cerebral and testicular tissues. As previously described, the 17β-estradiol (E2) has an important role in estrogen-dependent breast cancer, and it is described as a potential mutagenic and carcinogenic mediator [38]. Furthermore, its metabolites are also considered inducers of cell proliferation.

During their catabolism, AAS reveal their oxidative role, increasing ROS, which are highly unstable and extremely reactive oxygen species, which easily lose hydrogen atoms. In this manner, they form covalent bonds with DNA bases or sequences, inducing a known genetic damage [39]. This mechanism was supposed to be connected to hepatocellular alterations because of its interference with bilirubin metabolism and vascular and cellular hyperplasia. For this kind of tumorigenesis action, different patterns of evolution were described, such as hepatic peliosis and focal-nodular hyperplasia/liver adenomas [40–41].

Moreover, in a study conducted by Seraj *et al.* [42] about testosterone derivative genotoxicity, its ability in generating DNA adducts was established. These processes, individually or in combination, can induce micronuclei formation among animals exposed to higher concentrations of AAS. Micronuclei are strictly related to several mutagenic stresses and are formed following chromosomal damage. These are chromatin particles derived from acentric chromosomal fragments, which are not incorporated into the daughter nucleus after mitosis. A variety of factors influences micronuclei formation, such as; age, sex, genetic constitution, physical and chemical agents. One of these agents has been proven high-dose nandrolone exposition, which determines a significant DNA damage in blood, liver, bone marrow, brain and testicle cells in experimental animals exposed [43]. On the other hand, AAS can elicit profound modifications in genetic sequences by means of alterations in telomerase activity [44]. Nourbakhsh *et al.* [45], tried to verify the implications of androgens in ovarian carcinogenesis. They demonstrated that both testosterone and androstenedione increased ovarian cancer cell viability via the expression, activity, and phosphorylation of telomerase, and by blocking phosphatidylinositol 3-kinase pathway inhibitors. Consequently, it was stated that the *PI3K/Akt* pathway triggered this upregulation and that the *hTERT* mRNA levels were significantly increased with exposure to testosterone and androstenedione, thanks to quantitative PCR analyses. *hTERT* is a gene capable to encode the telomerase catalytic subunit, thus, allowing sustained cell proliferation, which has already been revealed to be regulated in human prostate cancer cells *in vivo* by androgens. In addition, androgen use has been reported to improve telomerase expression and behavior. Tamoxifen and letrozole inhibit estradiol and androgens effects on telomerase activity which is not affected by flutamide (an androgen receptor antagonist) administration [46]. Alterations in telomerase activity are at the basis of stanozolol-induced DNA-damaging effects.

With regards to the theoretical genotoxic effects on DNA, it should be pointed out that AAS effects are linked to dosage and frequency of administration. Therefore abusers abide by strict and controlled administration regimens resorting to specific strategies. “Stacking”, one of the strategies employed, involves the use of multiple AAS in order to lower doses of each substance and their adverse effects [47]. A common occurrence is the simultaneous administration of AAS and growth hormone (GH). It is estimated that one in each four sportsman takes both these drugs [48–49].

**Figure 2:**
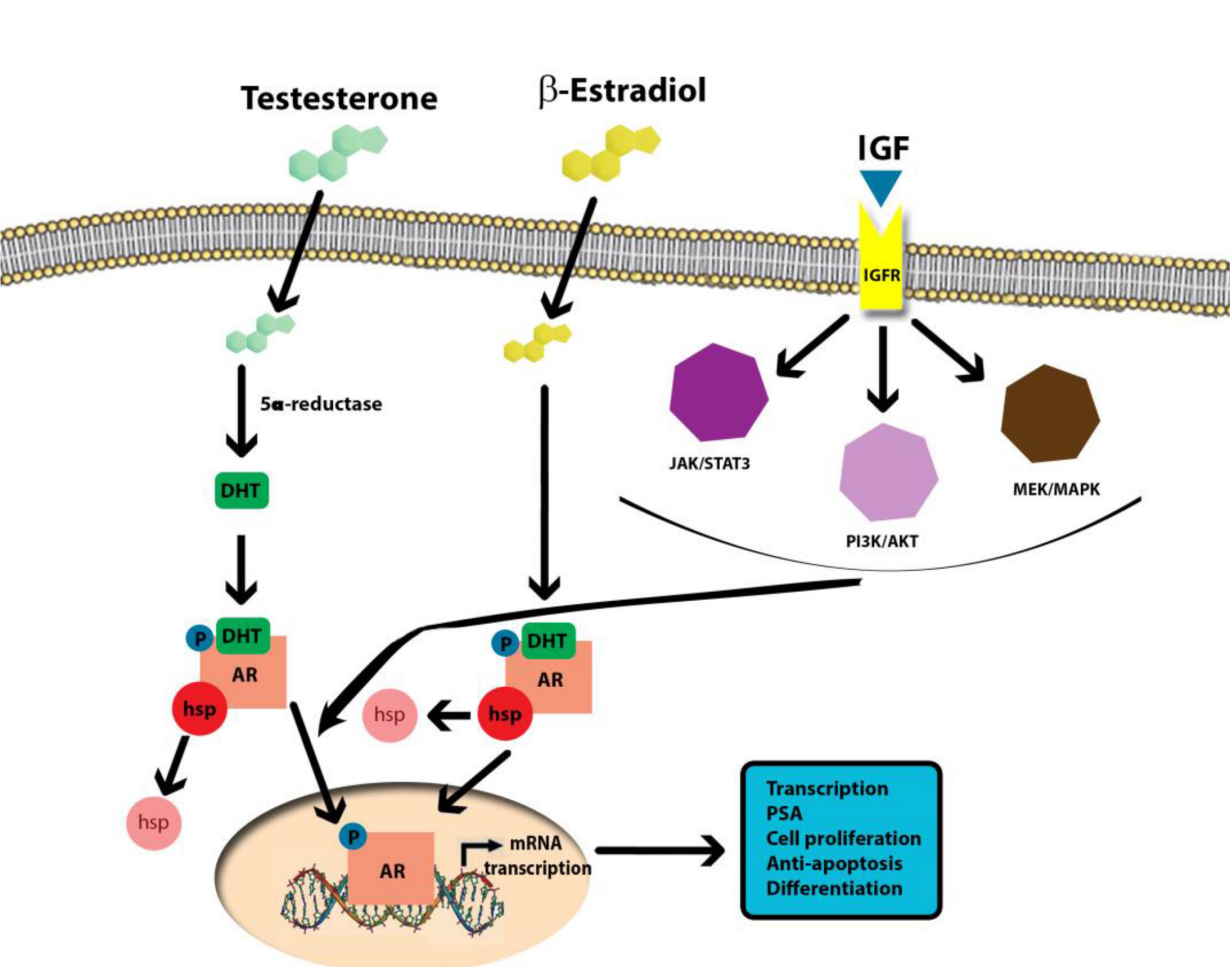
Molecular mechanisms of AAS-induced carcinogenicity

AASs promote muscle fiber mass and hypertrophy by augmentation of satellite cell proliferation, myonuclei number and muscle protein synthesis [50]. GH, instead, directly regulates muscle protein expression and production, by binding its receptors but also indirectly by activation of the *IGF-1* receptor, which can activate the *PI3K/Akt* pathway in order to endorse myocellular proliferation (Figure 2). As far as myogenesis is concerned, *IGF-1* is a positive key signaling molecule inducing not also satellite cell multiplying but also myoblast differentiation and subsequent myoblast fusion into myotubes. Consequently, overexpression of *IGF-1* causes significant hypertrophy and excess in proliferation [51]. Furthermore, *IGF-1* seems to mediate the growth-promoting influences of anabolic steroids. Based on that, AAS and *GH or IGF-1* are combinations with a high performance-enhancing potential. Testosterone stimulates the mitotic activity of satellite cells, while *GH or IGF-1* mediates these effects on skeletal muscle cell growth and differentiation [52]. This combined effect on myocells, should be considered in the light that supra-physiological doses of GH are associated with increased incidences of colorectal, thyroid, breast, and prostate cancers. Moreover, high-level detection of plasmatic *IGF-1* has been associated with cancer risk and cancer prognosis [53]. *IGF-1* has been involved in tumor development and progression because cancer cells have an increased expression of *IGF-1* receptors. In addition, Cappello *et al.* [54] have demonstrated that Heat shock proteins (Hsps) perform critical functions in maintaining protein homeostasis under physiological and stressful conditions. *Hsp60* is a constitutive mitochondrial protein with specific functions related to mitochondrial protein folding, especially in response to oxidative stress [55–56]. *Hsp90* was found to be overexpressed in multiple cancers, including prostate cancer. *Hsp90* is able to activate over 200 proteins, including the AR, where *Hsp90* prevents proteasome degradation and stabilizes AR conformation poised for ligand activation [57]. AR binds to *Hsp90* in its inactive form. Upon binding of androgens, the receptor detaches from *Hsp90* and becomes activated [58–60].

Sirianni *et al.* [61] using a human breast cancer cell line, *MCF-7*, as an experimental model, demonstrated that stimulating aromatase expression and estrogen production through *IGF-1* can promote cell proliferation. High doses of nandrolone (aromatizable) and stanozolol (non aromatizable), could potentially increase breast cancer risk because in cases of high bioavailability, these compounds can attach to the *ER*, inducing its nuclear translocation *in vivo* [62–64]. Rapidly increase in the concentration of *IGF-1R, ERK1/2* and *AKT* phosphorylation, is demonstrated by the ICI block on AAS-dependent kinase activation. Considering the above data, estrogens induce cell proliferation in target cells via the increased expression of up-regulated *CCND1*, encoding cyclin D1 regulating cell cycle G1 phase, which determines breast cancer cell proliferation [65–67]. Nandrolone and stanozolol magnified cyclin D1 concentration, inducing cell proliferation.

## TESTICULAR CANCER

Testicular cancer represents 1% of male neoplasms and 5% of urological tumors, with 3–10 new cases occurring per 100,000 males/per year in Western society [68–69]. In the last decades, the incidence of this cancer is constantly increasing, especially in industrialized countries [70–72]. Leydig cell tumors are usually benign, but approximately 10% are malignant. The malignant variants occur only in adults.

Table 1 subdivides testicular cancers according to a pathological classification, in germ cell tumors, sex cord/gonadal stromal tumors and miscellaneous non-specific tumors [73].

**Table 1:** Subdivision of testicular cancers under pathological classification

Germ cell tumors	Sex cord/gonadal stromal tumors	Miscellaneous non-specific tumors
• Intratubular germ cell neoplasia (IGCNU), unclassified type • Seminoma (including cases with syncytiotrophoblastic cells) • Spermatocytic seminoma (mention if there is a sarcomatous component) • Embryonal carcinoma • Yolk sac tumour • Choriocarcinoma • Teratoma (mature, immature, with malignant component) • Tumours with more than one histological type (specify percentage of individual components).	• Leydig cell tumour • Malignant Leydig cell tumour • Sertoli cell tumour - lipid-rich variant - sclerosing - large cell calcifying • Malignant Sertoli cell tumour • Granulosa cell tumour - adult type - juvenile type • Thecoma/fibroma group of tumours • Other sex cord/gonadal stromal tumours - incompletely differentiated - mixed • Tumours containing germ cell and sex cord/gonadal stromal (gonadoblastoma)	• Ovarian epithelial tumours • Tumours of the collecting ducts and rete testis • Tumours (benign and malignant) of non-specific stroma.

Leydig cell tumors represent the most common kind of the sex/gonadal stromal category [74–75]. Their insurgency is located between the third and sixth decades of adult life: when it occurs before, it is frequently related to hormones therapy/abuse.

Adults with androgen-secreting tumors are generally asymptomatic. Adult clinical manifestations of estrogen-secreting tumors include loss of libido, erectile dysfunction, infertility, gynecomastia, feminine hair distribution, and gonadogenital atrophy [76]. Taking into account the etiology, Leydig cell tumors are associated with cryptorchidism, testicular atrophy, infertility, germline mutations in fumarate hydratase, hereditary leiomyomatosis and renal cell carcinoma [77]. These symptoms are commonly found in AAS abusers. In fact, the testicular atrophy represents one of the most frequent side-effects related to AAS abusers, so a relationship between Leydig cell cancer and AAS must be taken into consideration.

## AASS AND LEYDIG CELL CANCER

To investigate the side effects of AASs abuse on testicular cells, several animal studies were performed. Dohle *et al.* [78] showed that exogenous administration of synthetic testosterone caused a negative alteration on the hypothalamic-pituitary axis, inhibiting the secretion of both Follicle-stimulating hormone (FSH) and luteinizing hormone (LH). This mechanism could lead to a decreased serum androgens concentration and cause hypogonadotropic hypogonadism with subsequent testicular atrophy. Androgen action is mediated by binding to androgen receptors (AR) both in testis and in other tissues [79]. Inside Sertoli cells, the receptor activation will represent the start impulse of spermatogenesis [78]. In rats’ testis, ARs are expressed in the somatic Leydig cells, in peritubular myoid cells, and in Sertoli cells as well as in rete testis, the epithelial cells of the epididymis, and prostate [80]. AASs effects are strictly dependent on AR presence and distribution, suggesting an AASs influence on the male reproductive system.

AASs abuse induces testicular damage by triggering oxidative stress via inflammatory cytokines, matrix metalloproteinases, cell adhesion molecules, apoptotic markers, and DNA damage [81–83]. These mechanisms interfere with testis development, morphology, function, and sperm features. In this context, Noorafshan *et al.* [84] showed that weight and volume of testis decreased in animals that received high doses of AASs in comparison to the control animals. Other studies, performed on animal models confirmed that AASs administration in animal model induced testicular weight reduction [85]. Furthermore, it is well described that in animal model AASs assumption can cause morphological changes, such as reduction of number and size of Leydig cells, cytoplasmic vacuolization, on seminiferous tubule length and lipid droplet deposition [81, 83–84]. Moreover, several sperm alterations were observed in animals undergoing AASs administration. Treatment with relatively high doses of AASs leads to a decrease sperm count, and sperm motility [84, 86–89]. Both high and low doses of AASs significantly lowered the sperm motility compared to the control group. These findings suggest relevant sperm alterations due to AAS abuse. Finally, it is well known that anabolic agents may induce cell proliferation. Several studies reported the relationship between AASs assumption with infertility and carcinogenesis progression [90–91].

Barone *et al.* [92], demonstrated blood–testis barrier (BTB) alterations. Tight junctions (TJs), basal ectoplasmic specializations (ES) and desmosome–gap junctions (D-GJs) compose the BTB in order to distinguish the basal and adluminal compartments in the seminiferous tubules. This study showed an increase in gene coding of TJ-integral membrane protein adaptor, *TJP1* and an abnormal distribution with immunofluorescence staining was revealed in cell cytoplasm of the basal compartment and in some cells at the adluminal compartment. *TJP1* anomalous localization has been linked to epithelial carcinoma *in situ* [93–96]. Furthermore, an altered induction of *MUC1* was suspected after nandrolone administration. This protein is a component of the mucosal glycocalyx associated with the testicular germ cell line and impaired spermatogenesis [97–98]. Usually intracytoplasmic, *MUC1* expression was detected by Barone *et al.* [92] in the nuclei of many seminiferous tubule spermatids in different mice treated with different nandrolone doses. *MUC1* nuclear translocation from the cytoplasm has been associated with transcription control and cell proliferation, mimicking an oncoprotein [91, 99–100].

Using rat Leydig tumor cell line, *R2C* cells, as an experimental model, Sirianni *et al.* [101], demonstrated, that high concentration of androgens promotes Leydig cell aromatase metabolism, determining the presence of local estrogen quantities. The resulting aromatase over-expression in this tumor cells leads to considerable higher-than-normal E2 levels, which can either initiate or cause progression of Leydig cell tumor.

This mechanism is mediated differently by different AAS classes, as shown by the comparison of nandrolone, an aromatizable androgen, with stanozolol, a non-aromatizable one. Stanozolol is not susceptible to aromatase metabolism, resulting in a high concentration of testosterone, which provokes aromatase over-expression, transcription and production. Nandrolone is easily and rapidly converted to estradiol, because of constitutively aromatase activation in Leydig *R2C* cells. This causes an immediate increase in estrogen quantities with less androgen concentration, promoting aromatase gene, *CYP19*, transcription. In an experimental animal model also conducted on *R2C* cells, Pomara *et al.* [102] reported that testosterone levels increase when lower nandrolone concentration are administrated to Leydig cells. The increment stopped after treatment with higher androgenic concentrations. Different dose-dependent effects are caused by the nandrolone-induced modification in genetic expression in testosterone synthesis molecules, in particular, steroidogenic acute regulatory protein (*StAR*) and *CYP17A1*. The exact mechanism by which nandrolone exerts its effect is currently not known yet, but it could possibly be through miRNA regulation, post-translational modification or protein degradation.

Nandrolone is an androgen receptor agonist. On binding to the AR receptor, it may induce the release of the AR receptor from *Hsp90* and its translocation to the nucleus [103]. In our previous experiments, higher nandrolone concentrations induced a more pronounced increase in *Hsp90* levels of expression and phosphorylation. This result is an indirect demonstration that nandrolone binds to AR-receptor and induces its activation. AR-receptor may act as a transcription factor binding to *HREs* or rapidly activating the MAP kinase pathway; and activates the *CREB* transcription factor via phosphorylation of *ERK1/2* or through the direct binding of *CREB* in the cytoplasm [104]. The expression of *STAR, HSD3B1, CYP11A1, CYP17A1* as well as of other enzymes of the testosterone biosynthesis pathway is activated by cAMP steroidogenesis. The latter is activated by the MAP kinase and *CREB* pathway, which is triggered by the non-conventional activation of the AR-receptor. In our experiments, nandrolone induced over-expression of STAR and *HSD3B1*, but downregulated *CYP17A1* and *CYP11A1*. The down-regulation of *CYP17A1* and *CYP11A1* can be explained as an effect of nandrolone binding to the AR while the overexpression of STAR and *HSD3B1* is consistent with the progesterone-like activity of nandrolone, overall leading to a decrease of testosterone synthesis [105].

Thus, Chimento *et al.* [106], suspected that pathophysiology of AAS-induced cancer induction and proliferation depends on two mechanisms. Firstly, the so-called estrogen-dependence that is related to estrogen production and not related to their androgenic effect. Secondly, the IGF-1R implication: this complex process was apparently demonstrated through the observation that ICI, an ER antagonist, reduces AAS-dependent cell proliferation. The same effect is generated by administration of IGF-1R pathway inhibitors (including IGF1R, PKC and PI3K inhibitors) which provoke a decrease of aromatase expression.

This consideration is not very innovative, considering that previous scientific literature extensively reported the estrogenic actions of androgens and the IGF-1R activation secondary to the binding of AAS to a membrane ER [107–108]. In human primary prostatic stromal cell cultures, DHT and T have already reported influencing *IGF-1* protein expression. This was not the case E2 [109].

Whatever the underlying mechanism is (either a non-genomic AAS molecular process involving *IGF-1* dependent signaling pathways or AAS-activated IGF-1R signaling through a membrane AR) the result is an activation of the *IGF-1*-mediated cascade pathway.

In turn, *IGF-1* signaling activation of *PI3K/AKT* and *PLC/PKC* pathways, results in an increase in aromatase expression and estrogen production inducing cell proliferation in Leydig cell cancer [101].

## DISCUSSION

AASs are a known cause various functional disorders, which may affect liver, cardiovascular, reproductive, musculoskeletal, endocrine, renal, immunologic or neuropsychiatric systems. Their effect is related to dosage, frequency, and patterns of use [110–111]. Beyond these deleterious macro-effects, testosterone-derived androgens may act directly on cellular functions, with either genetic or epigenetic factors determining toxic, mutagen, genotoxic and carcinogenic results. AAS can also influence cancer cell proliferation via genomic and non-genomic mechanisms, such as the so-called estrogen-dependence mediated by *ER*, aromatase expression and *IGF-1* production, which can even amplify each other. Therefore, *IGF-1* can be considered as a well-known cancer inducer and promoter affecting each stage of tumor development, from cellular proliferation to the metastatic phase.

In the study conducted by Chimento *et al.* [106], aromatizable and non-aromatizable androgens promoted testicular tumor development in rat Leydig cells via the pathophysiology described above. This suggests that further and analogous consideration to human Leydig cell cancer should be considered. However, these are not the only mechanisms suspected to be involved in cancer development after AAS use. Barone *et al.* [92], analyzed alterations that occur at the blood–testis barrier (BTB). They reported modifications induced on Leydig cells different from those induced by *IGF-1*. This study showed an increase in gene coding of TJ-integral membrane protein adaptor, *TJP1* and an anomalous localization linked to epithelial carcinoma *in situ* [93–96]. Furthermore, after nandrolone administration, *MUC-1* nuclear translocation has been associated with transcription control and cell proliferation, mimicking an oncoprotein [91,100].

Other mechanisms hypothesized involved DNA-damage at different levels include: micronuclei formation [34, 42], DNA methylation [36], ROS-mediated DNA covalent binding [39], and alterations in telomerases [44–45].

## CONCLUSIONS

The negative role of AAS in supraphysiological dosage impairs the expression of enzymes involved in testosterone biosynthesis. The side effects on the natural synthesis of testosterone play a potential role on the hormonal changes/regulation and could be suspected to be at the base of certain carcinogenic mechanisms. Furthermore, easily accessible and commonly diffused AAS, such as nandrolone and stanozolol, have the potential to induce and cause progression of particular kinds of cancer, such as Leydig cell tumor through multiple processes pathways. Their deleterious effect is further augmented by the fact, that such tumors have a high incidence in young people, the cohort of people abusing AAS. Further studies are necessary to investigate the potential link between AAS abuse and cancer.
